# Effects of *Maerua subcordata* (Gilg) DeWolf on electrophile-responsive element (EpRE)-mediated gene expression *in vitro*

**DOI:** 10.1371/journal.pone.0215155

**Published:** 2019-04-15

**Authors:** Mebrahtom Gebrelibanos Hiben, Laura de Haan, Bert Spenkelink, Sebas Wesseling, Jochem Louisse, Jacques Vervoort, Ivonne M. C. M. Rietjens

**Affiliations:** 1 Division of Toxicology, Wageningen University, WE Wageningen, The Netherlands; 2 Department of Pharmacognosy, School of Pharmacy, College of Health Sciences, Mekelle University, Mekelle, Ethiopia; 3 Laboratory of Biochemistry, Wageningen University, WE Wageningen, The Netherlands; ICAR-Indian Institute of Agricultural Biotechnology, INDIA

## Abstract

Plant extracts and phytochemicals may prevent chronic diseases *via* activation of adaptive cellular stress response pathways including induction of antioxidant and phase II detoxifying enzymes. The regulatory regions of these inducible genes encode the electrophile-response element (EpRE). This study tested the EpRE induction ability of *Maerua subcordata* (fruit, leaf, root, seed) methanol extracts and selected candidate constituents thereof, identified by liquid chromatography coupled with multistage mass spectroscopy, employing an EpRE luciferase reporter gene assay using hepa-1c1c7 mouse hepatoma cells. A parallel Cytotox CALUX assay using human osteosarcoma U2OS cells was used to monitor any non-specific changes in luciferase activity or cytotoxicity. Results showed that fruit, root, and seed extracts were non-cytotoxic up to a concentration of 30 gram dry weight per litre but the leaf extract exhibited some cytotoxicity and that the leaf (despite some cytotoxicity), fruit, and seed extracts showed strong induction of EpRE mediated gene expression while induction by the root extract was minimal. Selected candidates included glucosinolates, isothiocyanates, and some biogenic amines. Subsequent studies showed that methyl-, ethyl-, isopropyl-, isobutyl- isothiocyanates, and sec-butyl thiocyanate as well as glucobrassicin induced concentration (1–100 μM) dependent EpRE-mediated gene expression while the biogenic amines stachydrine and trigonelline acted as inhibitors of EpRE-mediated gene expression at 100 μM. The identification of glucolepidiin, glucobrassicin, glucocapparin, stachydrine, and trigonelline in all extracts was confirmed using standards and based on multiple reaction monitoring; yet, glucobrassicin level in the root extract was negligible. In conclusion, this study provided a first report on EpRE mediated gene expression effects of *M*. *subcordata*; and despite detection of different glucosinolates in all extracts, those containing glucobrassicin particularly displayed high EpRE induction. Because EpRE inducers are cytoprotective and potential chemopreventive agents while inhibitors are suggested adjuvants of chemotherapy, results of this study imply that process manipulation of this plant may result in herbal preparations that may be used as chemopreventive agents or adjuvants of chemotherapies.

## Introduction

Compelling scientific evidences revealed an inverse relationship between consumption of plant foods rich in some phytochemicals and the risk of several chronic diseases [1–5]. Although the basic protective mechanism has not been clearly explained yet, one suggested mechanism is that phytochemicals activate adaptive cellular stress response pathways. While a multitude of cellular targets are affected by phytochemicals, upregulation of the nuclear factor erythroid 2-related factor 2 (Nrf2) pathway could be a key factor in the cytoprotective properties of phytochemicals by means of which they promote cellular adaptation [1, 6–9].

Under homeostatic conditions, Nrf2 binds to Kelch-like ECH association protein 1 (Keap1), a cytosolic repressor protein that retains it in the cytoplasm and promotes its proteasomal degradation [8,10]. In the presence of Nrf2 activators (oxidative stress, some chemicals including phytochemicals), the Nrf2 dissociates from Keap1 and translocates in to the nucleus where it assembles to a cis-acting regulatory sequences called antioxidant response element (ARE) or electrophile-response element (EpRE), located in the promoter region of genes encoding various antioxidant and phase 2 detoxifying enzymes and resulting in their expression [8,10]. The main mechanism of Nrf2 activation is Keap1-dependent but Keap1 independent mechanisms such as Nrf2 phosphorylation are also reported [11]. Several reactive cysteine residues in Keap1 function as sensors of cellular redox changes and oxidation/covalent modification of some of these critical cysteine thiols in Keap1 would hamper its role in Nrf2 degradation thereby stabilizing Nrf2 and facilitating its nuclear accumulation [8, 10–12]. It is also worth mentioning that prolonged expression of Nrf2 was shown to protect cancer cells by inducing the metabolism and efflux of chemotherapeutics, leading to both intrinsic and acquired chemoresistance to anti-cancer drugs, an effect that can be regarded as the "dark side" of Nrf2. Thus, Nrf2 inhibitors have been suggested as adjuvant therapies to sensitize cancers with high expression of Nrf2 [13] or as treatment options of chemo- and radio-resistant forms of cancer [14].

*In vitro* assays are becoming more attractive as screening tools because they are rapid, and they have the potential to reduce the number of animals needed for chemical testing [15,16]. Among cell-based reporter gene assays, luciferase assays are often the method of choice for high-throughput screening for many reasons, most notably the high signal above background inherent to bioluminescence [17,18]. Stably transfected cell lines offer several advantages compared to other *in vitro* systems as they are an excellent aid in defining the mechanisms of action of unknown compounds [15]. *Maerua subcordata* (Gilg) DeWolf (Capparidaceae) is a wild famine food and medicinal plant mainly grown in the dry parts of East Africa where especially its root tuber and leaf parts have many ethnomedicinal claims including treatment of infectious diseases, malaria, ophthalmic and respiratory problems, allergic disorders, wounds, gastrointestinal disorders, pain, diabetes, and blood pressure. It is also used as a tonic agent, appetizer, as well as to induce sleep (high dose) and abortion [19–25]. Thus, considering the above viewpoints and that various plants are a very rich source of phytochemicals that activate the Nrf2 transcription factor [7,10,26], the aim of the present study was to evaluate, using the EpRE-LUX assay and LC-MS^n^ metabolic profiling, if *M*. *subcordata* extracts may activate the Nrf2 pathway and identify possible phytochemicals responsible for the activation.

## Materials and methods

### Chemicals and reagents

Arecaidine hydrochloride was purchased from Alfa Aesar (Karlsruhe, Germany); N-acetylagmatine from Cayman Chemicals-Europe (Sanbio Uden, the Netherlands); sinigrin potassium salt and glucolepidiin potassium salt were from Extrasynthese (Genay Cedex, France); glucobrassicin potassium salt, stachydrine hydrochloride, and trigonelline hydrochloride were from PhytoLab (Vestenbergsgreuth, Germany); agmatine sulfate, dimethyl sulfoxide (DMSO), glucosinolate hydrolysis products (methyl-, ethyl-, isobutyl-, isopropyl-isothiocyanates, and sec-butylthiocyanate), pipecolic acid, tert-butylhydroquinone (tBHQ), Viscozyme L, and resazurin sodium salt were from Sigma–Aldrich (Schnelldorf, Germany/ Zwijndrecht, The Netherlands). Minimum Essential Medium alpha (α-MEM), Minimum Essential Medium alpha 1:1 mixture of Dulbecco’s modified Eagle’s medium and Ham’s F12 medium (DMEM/F12), foetal calf serum (FCS) and Phosphate Buffered Saline (PBS) were from Gibco life technology (Paisley, UK)_;_ trypsin, nonessential amino acids (NEAA), and G418 were purchased from Invitrogen Corporation (Breda, The Netherlands).

### Collection, authentication, and processing of plant material

The fruit, leaf, root tuber, and seed parts of *Maerua subcordata* were collected from Shiraro area of Tigray, Northern Ethiopia (**S1 Fig**). This wild plant, sometimes considered as invasive weed, does not raise concerns of endangered or protected species. Collection was made from unreserved and publically open locality where local healers also collect their target medicinal plants and hence no special permission was required to make the collection. The plant was authenticated and a specimen (Voucher number MG001/2007) deposited in the National Herbarium at Addis Ababa University, Addis Ababa, Ethiopia. The plant parts were sorted and dried in the laboratory of Pharmacognosy, Mekelle University, Mekelle-Ethiopia. The outer thin coating of the underground part (root tuber) was peeled off and the peeled tuber was chopped. The chopped pieces were allowed to dry in an oven at a temperature of 40 ^o^C for four days. The other parts (fruit, leaf, and seed) were dried in the shade at room temperature and fruits were deseeded (seeds taken out from fruits) after drying. The dried plant materials were packed in plastic bags, and stored at room temperature on shelf until they were transported to Wageningen University, the Netherlands; where they were powdered: each dried plant part was splashed with liquid nitrogen to remove moisture and facilitate powdering, and ground using an analytical miller. Each powdered plant material was mixed well, packed in capped plastic tubes, and stored at -80 ^o^C until further use.

### Cell lines

Cytotox CALUX and EpRE-LUX cell lines were used in the present study. The cytotox CALUX cells (BioDetections Systems, Amsterdam, The Netherlands) are human osteosarcoma U2OS cells stably transfected with a reporter construct carrying a luciferase reporter gene under transcriptional control of a constitutive promoter. These cells have an invariant luciferase expression and were originally designed to assess cytotoxicity. However, they can also be used to investigate whether stabilisation of the luciferase enzyme is occurring during the exposure to compounds or extracts, a phenomenon that would result in increased luciferase activity without underlying increased expression of the gene. These cells were cultured in DMEM/F12 supplemented with 7.5% (v/v) FCS, and 0.5% (v/v) NEAA [27,28]. EpRE (mGST-Ya)-LUX cells were previously developed based on EpRE sequences from the mouse glutathione-S-transferase Ya (mGST-Ya) gene [27]. These cells are hepa-1c1c7 mouse hepatoma cells stably transfected with a reporter construct carrying a luciferase reporter gene under transcriptional control of an EpRE-enhancer element in conjunction with a minimal promoter and an initiator [29]. The EpRE-LUX cells were cultured in α-MEM medium supplemented with 10% FCS and were maintained at 37 ^o^C in a humidified atmosphere with 5% CO_2_ [27,29]. For both cell lines, 200 μg/ml G418 was added to the culture medium once per week as selection pressure to maintain cells with constructs [27].

### Preparation of extracts from *Maerua subcordata*

Two types of extracts: non-hydrolysed and enzyme (Viscozyme L) hydrolysed extracts were prepared from powders of *M*. *subcordata* fruit, leaf, root tuber, and seed samples following the procedure described by Gijsbers *et al*. (2012) [27]. In short, non-hydrolysed extracts were prepared by adding 3.4 ml methanol to 0.6 g powdered plant material followed by 10 min sonication and 15 min centrifugation at 1000 g. Then the supernatant of each sample was filtered using 0.2 μm polytetrafluoroethylene (PTFE)-filters (Whatman, Germany) and freeze-dried after the methanol was evaporated under a stream of nitrogen. Dried extracts were stored at -80°C until further use. Prior to analysis in the EpRE-LUX assay, the extracts were re-dissolved in DMSO:α-MEM (1:2 v/v). Enzyme hydrolysed extracts were prepared by adding 300 μl sodium acetate (0.1 M, pH 4.8) and 100 μl of Viscozyme L to 0.6 g powdered plant material, followed by 1 hr incubation in a water bath at 37 ^o^C. Then, samples were put on ice and 3.0 ml of methanol was added to each sample. Subsequently, the samples were sonicated, centrifuged, filtered, dried and stored in the same way as described for the non-hydrolysed extracts. Prior to analysis in the EpRE-LUX assay these extracts were re-dissolved in DMSO:α-MEM (1:4 v/v).

### EpRE-LUX assay

The EpRE-mediated gene expression induction capacity of *M*. *subcordata* methanol extracts, selected individual compounds, tBHQ as positive control, and DMSO as solvent control was evaluated by measuring luciferase activity in EpRE-LUX reporter cells. The assays were performed essentially as described by Gijsbers *et al*. (2012) [27]. Briefly, EpRE-LUX cells were seeded in the 60 inner wells of a white 96-well view plate at a density of 20,000 cells per well in 100 μl culture medium. 200 μl PBS was added to the outer 36 wells to maintain physical homogeneity throughout the plate. The seeded cells were incubated for 24 hr to allow them attach and form a confluent monolayer. Then, the culture medium was carefully removed and, instantly, the cells were exposed to 200 μl α-MEM (without FCS and antibiotics) containing test samples for another 24 hr. The extracts were dissolved in DMSO:α-MEM (1:2 v/v) while tBHQ and the other individual compounds were dissolved in DMSO. The final concentration of DMSO in the exposure medium was 0.5% for studies with individual compounds whereas for studies with extracts, up to 1.5% was used after it was checked that 1.5% DMSO had no effect on cell viability compared to 0.5%. On each plate, 10 μM tBHQ was included as a positive control. After 24 hr exposure, medium was removed, cells were washed with ½ PBS (PBS half diluted with Nano pure water), and exposed to 30 μl low salt lysing buffer (1.2114 g Tris, 0.084 g dithiothreitol, 0.7287 g 1,2-cyclohexylenedinitrilotetraacetic acid; in a litre of Nano pure water, pH 7.8). Then plates were covered by aluminium foil, placed on ice for 15 minutes and frozen overnight at -80 ^o^C. Then the plates were thawed and luciferase activity per well in the lysate was measured in relative light units (RLU) using a luminometer (GloMax-Multi Detection System–Promega) and a flash mix (20 mM Tricine, 1.07 mM (MgCO_3_)_4_Mg(OH)_2_, 2.67 mM MgSO_4_.7H_2_O, 0.1 mM EDTA, 2.0 mM dithiothreitol, 470 μM luciferine, 5.0 mM ATP; in a litre of Nano pure water, pH 7.8). Results were expressed as fold induction compared to the solvent control. Extracts or compounds giving twofold or greater luciferase activity at a concentration that could be tested without cytotoxicity were considered able to induce EpRE-mediated luciferase expression.

### Cytotox CALUX assay

To each EpRE-LUX assay, a parallel test was done using the U2OS cytotox CALUX cells. These cells were seeded in the 60 inner wells of a white 96-well view plate at a density of 10,000 cells per well in 100 μl assay medium: DMEM/F12 supplemented with 7.5% (v/v) FCS, and 0.5% (v/v) NEAA. The outer wells were filled with 200 μl PBS. The next day, 100 μl medium containing the test samples was added to each well resulting in 200 μl assay medium per well. After 24 hr of exposure, luciferase activity was measured in the same way as in the EpRE-LUX assay described above.

### Alamar blue (resazurin) assay

Cytotox CALUX and EpRE-LUX cells were cultured in 96 well plates in their appropriate medium, described above, for 24 hrs and then the cells were exposed to test samples for another 24 hrs. Then alamar blue (resazurin) reagent solution (10% w/v in PBS) was added directly to the cells (10% v/v i.e. 20 μl reagent to 200 μl cells in medium). Following reagent addition, plates were covered by aluminium foil and incubated for 1 hrs after which fluorescence was measured (λex 580 nm/λem 610 nm) using a plate reader (Molecular Devices, Spectra Max M2) equipped with Softmax Pro software.

### LC−MS^n^ based metabolic profiling of *M*. *subcordata*

Liquid chromatography (LC) coupled with multistage mass spectroscopy (MS^n^) was applied to identify metabolites in methanol extracts from fruit, leaf, root, and seed of *M*. *subcordata*. For initial qualitative analysis, extracts were prepared by adding 400 μl of methanol (with 1% v/v formic acid) to 15 mg powder of each plant material. The mixture was vortexed, sonicated for 15 minutes, and centrifuged at 1000 g for 10 minutes. The supernatant was filtered using 0.2 μm polytetrafluoroethylene (PTFE)-filter (Whatman, Germany) and stored at -80 ^o^C until used. The extracts were subjected to LC-MS^n^ analysis based on coupling of ion-trap MS with Orbitrap Fourier transform MS. To support structural characterization of the many metabolites present in such complex samples, Ridder *et al*., (2012) [30] offered a novel method (http://www.emetabolomics.org/magma) to automatically process and annotate LC−MS^n^ data sets on the basis of candidate molecules from chemical databases. In 'Ms Annotation based on in silico Generated Metabolites' (MAGMa), which is an online application software, uploaded spectral data are automatically annotated with hierarchical trees of in silico generated substructures of candidate molecules, retrieved from PubChem and from a subset of PubChem compounds present in Kegg. Alternative structures of candidates are ranked on the basis of calculated matching score and displayed on the user interface [30,31]. The most likely candidate structures that could be possible constituents of the extracts were manually selected from the MAGMa user interface. The identity of selected candidates was further confirmed and quantified by LC-MS/MS based on multiple reaction monitoring (MRM) and calibration curves from standards. The identity of some candidates was further confirmed and quantified by standard calibration curves as described below.

### Confirmation of selected candidates by LC-MS/MS

This later analysis targeted mainly glucosinolates and extraction was done using a modified ISO9167-1 method as described by Ishida *et al*. (2011) [32] and Doheny-Adams *et al*. (2017) [33]. Briefly, into each test tube, 5 ml of 80% v/v methanol: water (buffered with 1% v/v formic acid) at room temperature (≈20 ^o^C) was added to 100 mg (dry weight) of powdered sample. The mixture was then vortexed and left for 30 min at room temperature. Then, it was ultra sonicated for 15 minutes and centrifuged at 1000g for 10 minutes. Supernatant was filtered (0.2 μm PTFE-filter) and stored at -80 ^o^C until used. LC-MS operating conditions were optimized using standard compounds and the extracts and the different concentrations of standard solutions for calibration curves were analysed under identical conditions. The MS analysis was performed in Multiple Reaction Monitoring (MRM) mode on LCMS-8040 (Shimadzu, Japan) triple quadrupole with Electrospray Ionization source operated in negative ion mode for glucosinolate analysis and positive ion mode for analysis of biogenic amines. The compounds of interest in the extracts were identified by their retention time and LCMS-MRM characteristics and quantified based on equations from calibration curves. Ares *et al* (2016) [34] used sinigrin as an external standard to quantify glucosinolates from broccoli where individual standards were not available. Likewise, sinigrin was used as external standard to quantify glucocapparin in *M*. *subcordata* extracts since a glucocapparin standard was not available.

### Data analysis

For each experiment, at least three independent repetitions were performed. Graphs generated from an average of repetitions are presented. Data were analysed using Microsoft Excel 2010/2016, expressed as fold induction over the solvent control, and presented as mean values ± standard error of the mean (SEM). Each data point was measured, at least, in triplicate. Statistical significance was assessed using SPSS 22, applying paired samples statistics t-tests and a significance cut-off value of p ≤ 0.05.

## Results

### Effect of *M. subcordata* methanol extracts on cell viability

**Fig 1:**
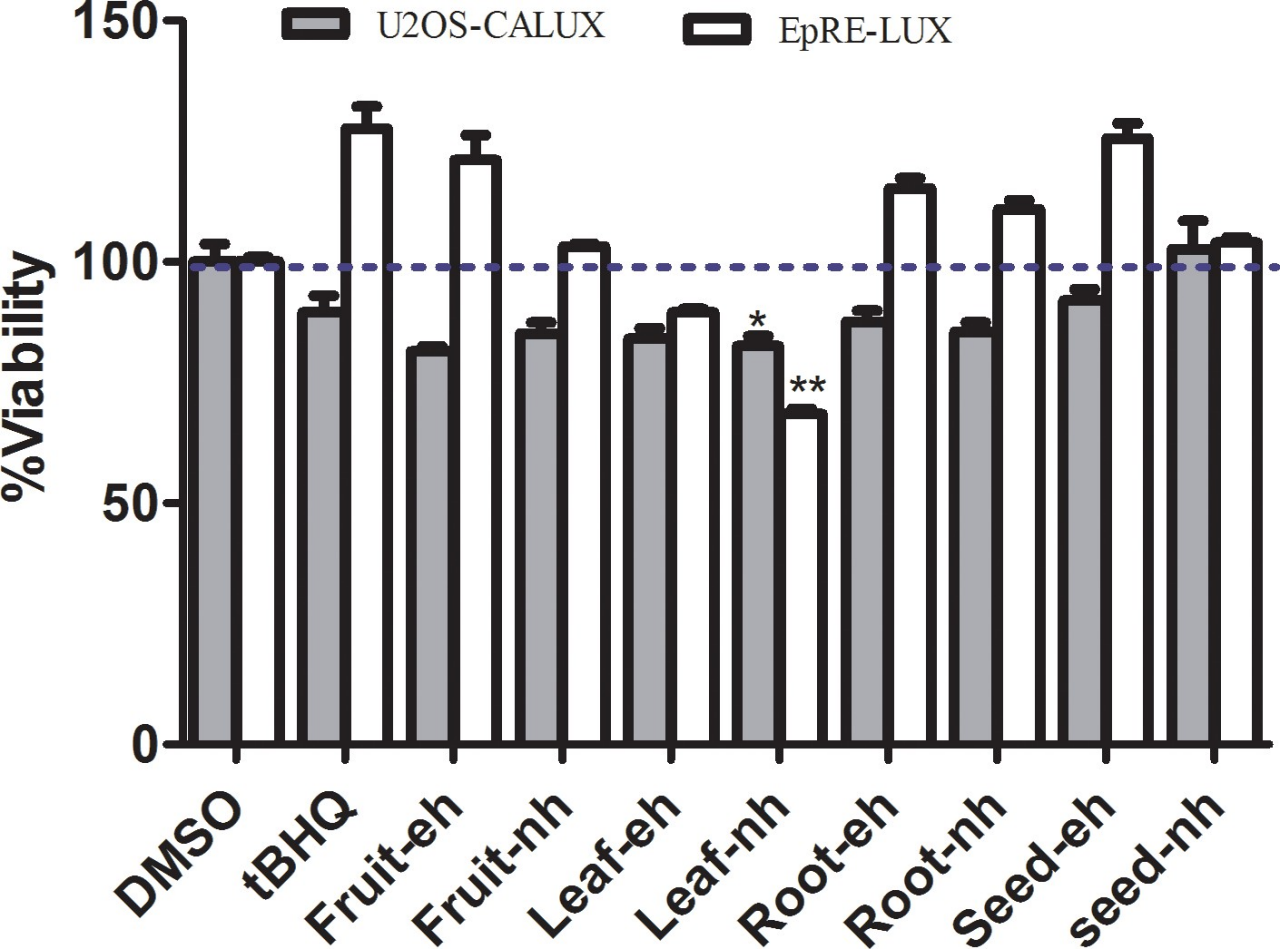
Resazurin assay results showing effects of enzyme hydrolysed (eh) and non-hydrolysed (nh) methanol extracts of different parts of *M*. *subcordata* at 30 gDW/L and tBHQ (10 μM) on cell viability. Data are presented as mean ± SEM of six replicates. Viability of cells exposed to the solvent control (1% DMSO) was set at 100% and asterisk indicate a significant difference from the solvent control: *p < 0.01; **p < 0.0001.

**Fig 2:**
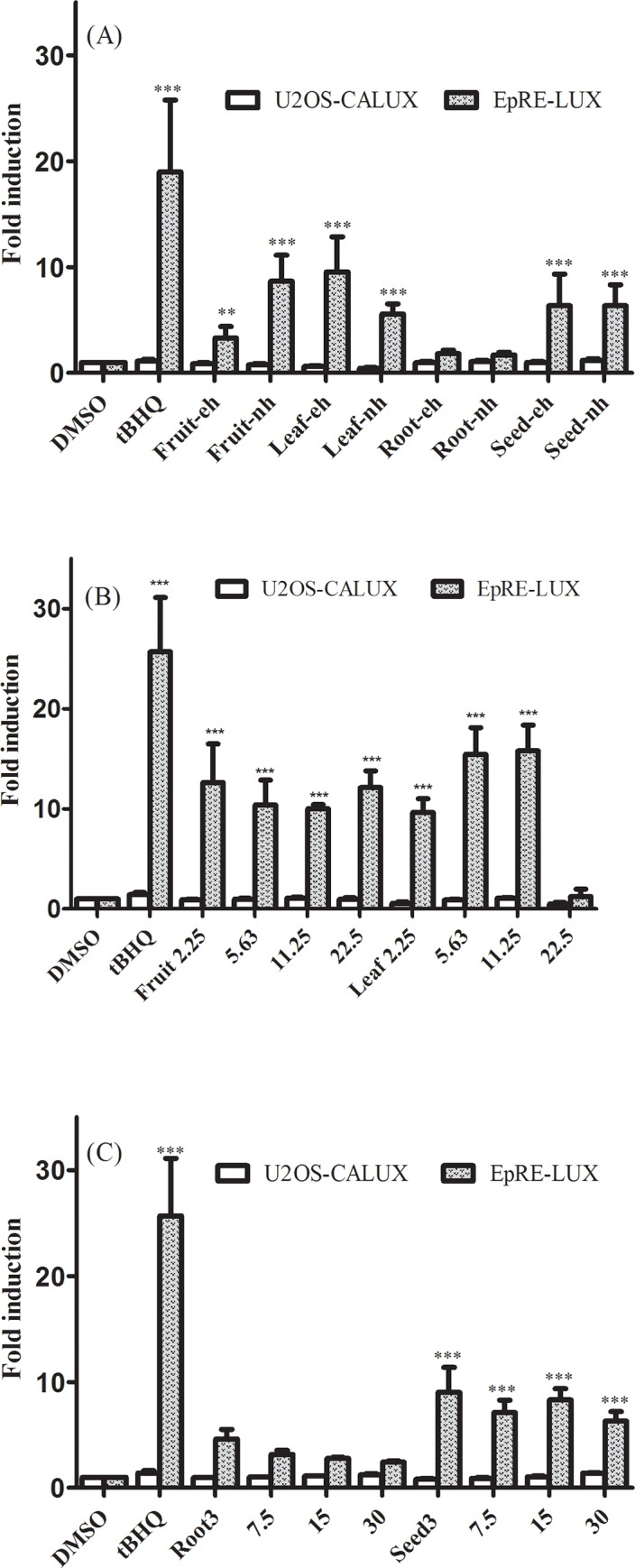
Luciferase activity in U2OS-CALUX and EpRE-LUX cells after 24 hr exposure to *M*. *subcordata* methanol extracts. (A) Enzyme hydrolysed (eh) and non-hydrolysed (nh) extracts at 30 gDW/L, different concentrations of (B) fruit and leaf nh extracts, and (C) root and seed nh extracts. Luciferase activity is expressed as fold induction compared to solvent control. tBHQ (10 μM) was used as the positive control. Data are presented as mean ± SEM of three independent experiments, each done in six replicates. Asterisks indicate a significant difference from the solvent control: **p < 0.01; ***p < 0.0001.

The resazurin assay results (**Fig 1**) show that the screened extracts up to a concentration of 30 gDW/L (gram dry weight per litre) cause no major effect on cell viability of both cell lines, except for the non-hydrolysed leaf extract that exhibited some cytotoxicity (68% and 82% remaining resazurin metabolic reduction activity for EpRE and U2OS cells, respectively). Also, testing with varying concentrations showed reproducible induction of EpRE mediated gene expression by the extracts (**Fig 2**) and the highest non-toxic concentration (30 gDW/L) of all extracts, except the leaf extract, was used as a screening concentration for all extracts so as to make comparison among them.

### Induction of EpRE mediated luciferase expression by *M. subcordata* methanol extracts

**Fig 3:**
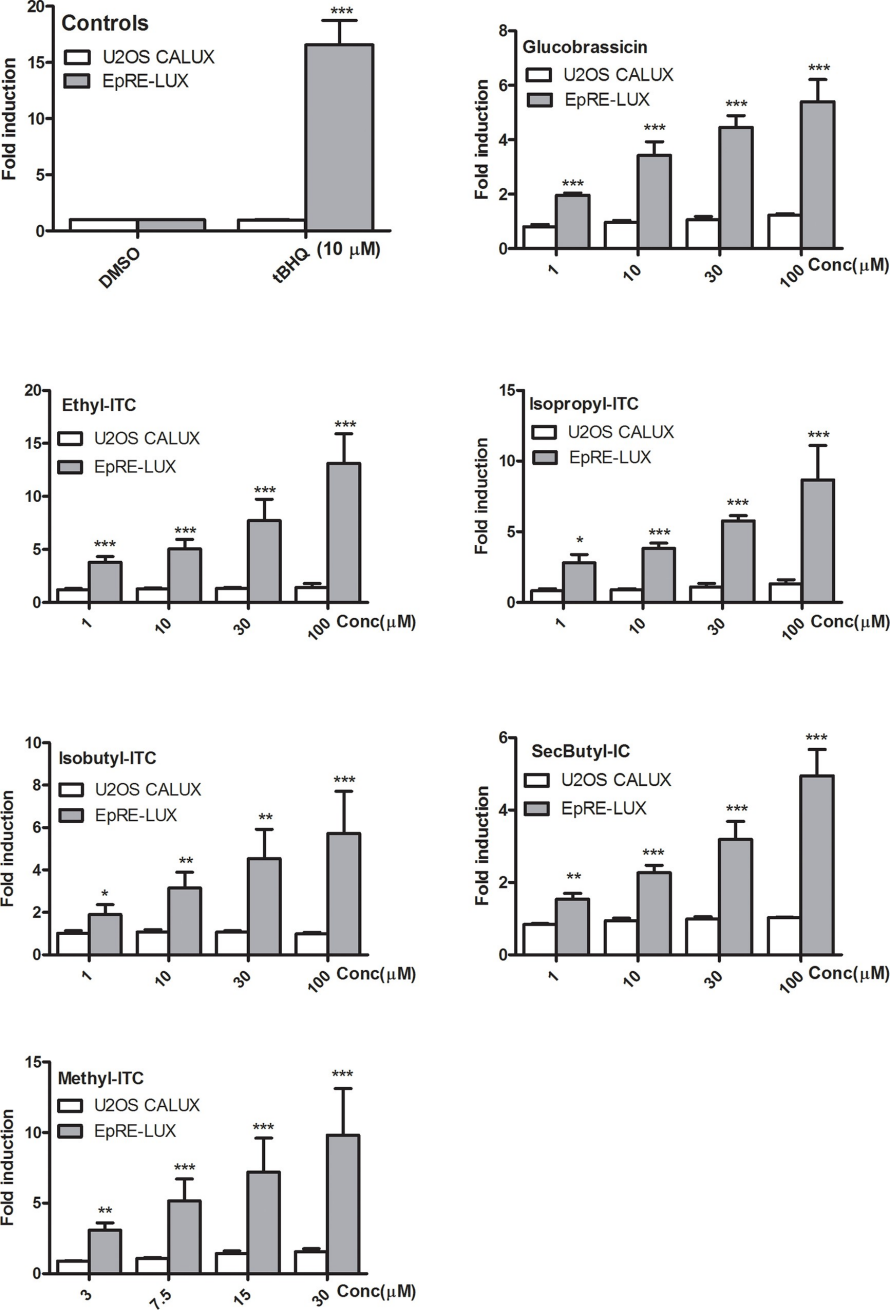
Induction of luciferase activity in U2OS and EpRE cell lines after 24 hr exposure to glucobrassicin, ethyl-, isopropyl-, isobutyl-, and methyl-isothiocyanates (ITCs), and sec-butyl isocyanate (IC). Luciferase activity is expressed as fold induction compared to the solvent control and data are presented as mean ± SEM of four independent experiments, each performed in triplicate. Asterisks show a significant difference from the solvent control: *p < 0.05; **p < 0.01; ***p < 0.0001.

The EpRE assay results show that both enzyme hydrolysed (eh) and non-hydrolysed (nh) methanol extracts from the fruit, leaf, and seed materials as well as tBHQ (positive control) exhibited a high increase in luciferase activity, whereas the induction by the root extracts was minimal as compared to the solvent control (DMSO) (**Fig 2A**). Unlike to what was expected, enzyme hydrolysis did not increase luciferase activity although enzyme hydrolysis seems to decreased the cytotoxicity of the leaf extract as the nh leaf extract was more cytotoxic than the eh leaf extract. Further assays with different concentrations of the non-hydrolysed extracts reproduced EpRE induction by the fruit, leaf, and seed extracts while the root extract showed some induction at lower concentrations that declined with increasing concentrations (**Fig 2B&2C**). These second round assays, which were performed along with candidate compounds, revealed that unlike the candidates that showed concentration dependent increase in luciferase activity (**Fig 3**), the extracts failed to maintain concentration dependent increase in luciferase activity. Moreover, although the maximum concentration for the fruit and leaf extracts was lowered to minimize cytotoxicity, the fruit extract was still non-cytotoxic but the leaf extract was even more cytotoxic at 22.5 gDW/L than previous assays done at 30 gDW/L implying high variability of the leaf extract both in cytotoxicity and induction of EpRE-mediated gene expression. Based on these observations the present report focused on the intrinsic properties of the extracts that the fruit, leaf, and seed exhibited reproducible high EpRE induction while induction by the root was minimal.

To monitor non-specific interference of luciferase by the extracts, a parallel test was done using the U2OS-CALUX assay. In line with minor effects on cell viability in the resazurin assay for the leaf extracts, the U2OS assay also shows that the leaf extracts exhibited some cytotoxicity (remaining luciferase activity of 62% and 44% compared to the solvent control for enzyme hydrolysed and non-hydrolysed, respectively) while the fruit, root, and seed extracts showed similar activity as the solvent control indicating the absence of cytotoxicity again in line with the resazurin assay. Since luciferase activity at non-cytotoxic concentrations in the U2OS-CALUX assays were similar as in the solvent controls, it was concluded that the extracts did not interfere with luciferase itself.

For fruit extracts, the non-hydrolysed extract showed greater EpRE induction than the enzyme hydrolysed while both type of seed extracts showed comparable induction. For the leaf, the enzyme-hydrolysed extract showed greater EpRE induction than the non-hydrolysed which could be due to a lower number of viable cells (44%) in the assay with the relatively higher cytotoxic non-hydrolysed extract. Although the leaf extracts were tested at levels that may induce some cytotoxicity, using the same concentration of 30 gDW/L allowed better comparison to the extracts from other plant parts to characterize their intrinsic properties towards EpRE-mediated gene expression.

### Induction of EpRE mediated luciferase expression by candidate compounds of *M. subcordata*

**Fig 4:**
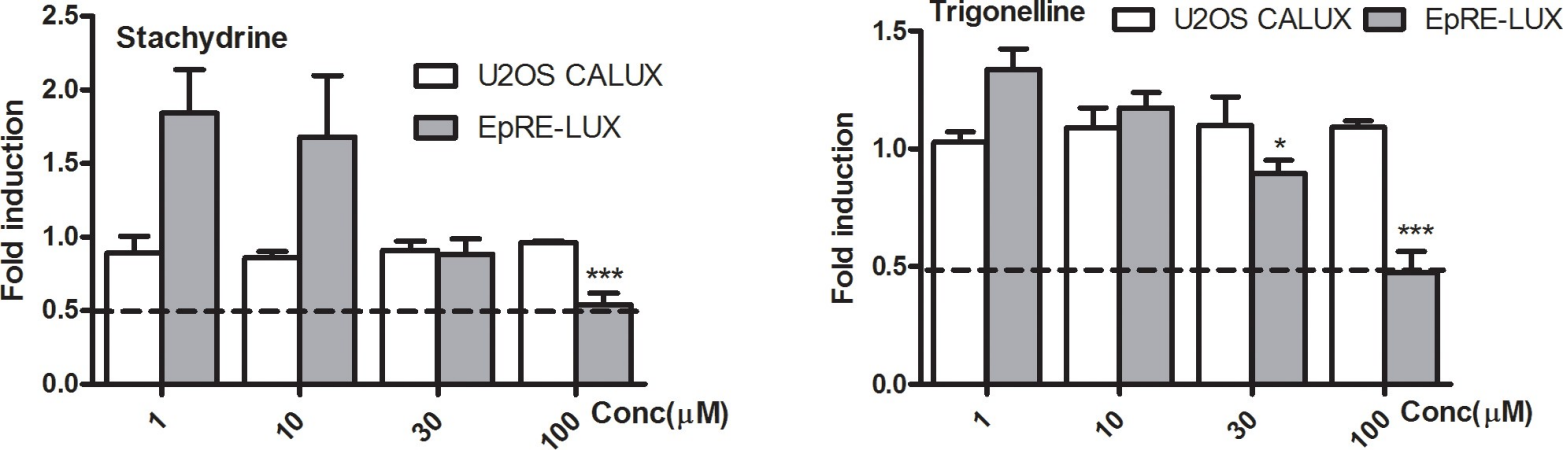
Luciferase activity induction in U2OS-CALUX and EpRE-LUX cell lines after 24 hr exposure to stachydrine and trigonelline. Luciferase activity is expressed as fold induction compared to solvent control. Data are presented as mean ± SEM of four independent experiments, each performed in triplicate. Asterisks show a significant difference from the solvent control: *p < 0.05; ***p < 0.0001.

To define the main phytochemicals responsible for the observed induction of EpRE mediated gene expression, LC-MS^n^ metabolic profiling was done on the methanol extracts. As enzyme hydrolysis did not influenced EpRE induction by the extracts to a large extent, only the non-hydrolysed samples were analysed. The LC-MS^n^ data resulted in a tentative identification of various constituents including glucosinolates (**S2 Fig)** and some biogenic amines (**S3 Fig)** to which focus was given as possible EpRE inducers. The glucosinolates were detected in negative ion mode while most biogenic amines in positive ion mode. Except for glucobrassicin, the respective isothiocyantes of the detected glucosinolates were screened for their EpRE mediated gene expression induction capacity because of their commercial availability. Thus, glucobrassicin, methyl-, ethyl-, isopropyl-, isobutyl- isothiocyanates, and sec-butyl thiocyanate which are hydrolysis products of glucocapparin, glucolepidiin, glucoputranjivin, isobutyl glucosinolate, and glucocochlearin respectively as well as some biogenic amines were tested in different concentrations (1–100 μM) for their EpRE mediated gene expression induction capacity. The results (**Fig 3)** showed that glucobrassicin, the isothiocyanates and *sec*-butyl isocyanate exhibited a concentration-dependent increase in luciferase activity compared to the solvent control. Conversely, the biogenic amines showed either no activity or a slight inhibitory effect on luciferase activity. The biogenic amines agmatine sulfate, N-acetylagmatine, anthranilic acid, pipecolic acid, and arecaidine hydrochloride were inactive up to a concentration of 100 μM while stachydrine and trigonelline showed inhibition (**Fig 4**) of EpRE mediated gene expression at the highest concentration tested (100 μM). None of these chemicals affected luciferase signal in the U2OS-CALUX assay up to the highest concentrations tested, indicating that they do not interfere with luciferase itself (**Figs 3 and 4**).

Further definite identification and quantification (**Table 1**) was made for glucolepidiin, glucobrassicin, stachydrine, and trigonelline by LC-MS/MS-MRM using standard calibration curves based on commercially available reference compounds. Moreover, glucocapparin was quantified using sinigrin as external standard^34^ since commercial reference glucocapparin was not available.

**Table 1:** Some glucosinolates and biogenic amines from *M*. *subcordata* extracts identified and quantified by LC-MS/MS MRM and standard calibration curves. (a) Amount described in microgram per gram dry weight (μg/gDW) and (b) extrapolated concentration (μM) in 96 well plate as calculated from a concentration of 30 gDW/L as applied in the *in vitro* studies with plant extracts. Rt, retention time.

*Samples*	Compounds									
	Glucocapparin		Glucolepidiin		Glucobrassicin		Stachydrine		Trigonelline	
***Rt (min)***	1.00		1.50		6.95		1.10		1.10	
	a	b	a	b	a	b	a	b	a	b
***Fruit***	154.69	13.92	1.44	0.12	2.07	0.138	2602.36	545.25	7.59	1.66
***Leaf***	539.61	48.56	8.86	0.76	28.72	1.921	3290.43	689.42	7.44	1.63
***Root***	390.60	35.15	13.05	1.13	0.06	0.004	1507.58	315.87	5.41	1.18
***Seed***	432.80	38.95	3.79	0.33	17.48	1.169	2710.23	567.85	6.53	1.43

One can speculate from **Table 1** that the glucosinolates, mainly glucocapparin may generate concentrations of matching ITCs that could explain a large part of the EpRE induction by all extracts including the root extract, if most would be hydrolysed to these ITCs. However, the root extract did not induce EpRE mediated gene expression. Another speculative view could be a possible antagonism by stachydrine as the extrapolated concentration of which is above 100 μM in all extracts. Yet, its level in the root is lower than in the other extracts. Therefore, despite the identification of components in the extracts at seemingly substantial levels that can influence EpRE mediated gene expression, it appears to be difficult to indicate the specific phytochemicals responsible for the EpRE induction by the extracts based on the current data. Further consideration of the complex interactions among multiple components in the extracts, the unpredictable kinetics of glucosinolate hydrolysis during the *in vitro* EpRE induction studies, and the contribution, on the overall effect, of other chemicals in the extracts that were not included in the chemical identification of the present study may be required. In this regard, an activity-directed analysis with fractions and sub-fractions could be of help to identify the phytochemicals with the largest effects. Interestingly, the fruit, leaf, and seed extracts that showed apparently high levels of glucobrassicin exhibited significant induction of EpRE mediated gene expression while the root extract which showed negligible glucobrassicin level, showed insignificant induction. This may indicate that glucobrassicin and possibly other related glucosinolates could be largely responsible for the induction of EpRE mediated gene expression exhibited by *M*. *subcordata* extracts.

## Discussion

The present study evaluated the EpRE mediated gene expression induction potential of *M*. *subcordata* methanol extracts and selected candidate compounds thereof, using an *in vitro* luciferase reporter gene assay. It was shown that the fruit, leaf, and seed extracts revealed strong induction of luciferase activity at 30 gDW/L while the induction by the root extract was less than twofold. To ensure that the induced increase of luciferase activity is not due to luciferase stabilization caused by non-specific or off-target interactions [17,18,35], a cytotox CALUX assay based screening was done parallel to each test with the EpRE-LUX assay [27,28]. Results of this counter screening showed luciferase activity of more or less similar to that of the solvent control for the fruit, root, and seed extracts at 30 gDW/L suggesting the absence of non-specific interference with the luciferase reporter protein. Significantly lower luciferase activity was shown by the leaf extract, which was in line with the reduction of cell viability as measured by the resazurin assay. The remaining cells exposed to the leaf extract at 30 gDW/L still showed a strong induction of luciferase activity in the EpRE assay. Enzyme hydrolysis with Viscozyme L was shown to improve both extraction and biological activity of phenolic compounds [27,36]. Expecting that *M*. *subcordata* may contain such constituents, enzyme hydrolysed (eh) extracts were prepared along with non-hydrolysed (nh) extracts. However, eh extracts did not show higher EpRE induction compared to nh extracts implying that the components responsible for EpRE induction by the extracts were not substrates of Viscozyme L. Indeed, results from the phytochemical analysis of the extracts revealed the presence of glucosinolates, known EpRE inducers, which are not substrates of Viscozyme L. Yet, enzyme hydrolysis seems to reduce cytotoxicity of the leaf extract (remaining viable cells luciferase activity of 62% and 44% for eh and nh, respectively) and hence a greater induction by the eh extract than the nh extract.

Liquid chromatography coupled with multistage accurate mass spectrometry(LC−MS^n^) can generate comprehensive spectral information of metabolites in crude extracts which contain complex multiple components. In the present study, structural characterization of metabolites in *M*. *subcordata* methanol extracts was done by LC−MS^n^−MAGMa structural annotation as described by Ridder *et al*., [30,31] that resulted in the tentative identification of many compounds including glucosinolates (GLs) and biogenic amines. Moreover, supporting literature data show that alkyl and indolyl GLs are constituents of many species of the Capparidaceae family including *Maerua* species [37]. There is no previous study reporting GLs in *M*. *subcordata*, but glucobrassicin, glucocapparin, and glucocleomin were reported in other *Maerua* species [38–40]. However, glucocleomin was not detected in any of the analysed extracts in the present study. Mainly, glucocapparin is widely distributed throughout the Capparidaceae [38,41] usually as the most abundant compound [40,42–45]. Likewise, glucocapparin was detected in the present study in all extracts of *M*. *subcordata* in apparently high amounts (**Table 1**) indicating that it also constitutes a major GL in *M*. *subcordata*.

Because GLs and their hydrolysis products are known inducers of Nrf2, they have been given focus as likely components responsible for the luciferase activity induction by *M*. *subcordata* extracts. Plants producing GLs possess a β-thioglucosidase, called myrosinase. GLs are stored in vacuoles of plant cells while myrosinases occur compartmentalized in separate but adjacent myrosin cells. Upon plant tissue damage, GLs get into contact with myrosinase at the damage site and hydrolysis is catalysed resulting in various products including isothiocyanates (ITCs), thiocyanates, and nitriles depending on reaction conditions such as pH and temperature. Hydrolysis at neutral conditions or pH 5–7 typically results in the formation of ITCs which are responsible for the biological activities of GLs [46–48]. GLs and their hydrolysis products, mostly ITCs, have long been known for their allelopathic, bacteriocidal, fungicidal, and nematocidal properties while recently, they attracted intense research interest because of their cancer chemoprotective attributes presenting a promising group of natural anti-infective and anti-cancer agents [37, 46, 48–50]. The interest on these unique phytochemicals with versatile biological properties have been more intensified after the discovery that the ITC, sulforaphane, potently induces mammalian cytoprotective proteins through the Keap1–Nrf2 pathway. Nowadays, it seems established that ITCs are well known to target Keap1 for activating Nrf2 pathway resulting in induction of gene expression of antioxidant and Phase II detoxifying enzymes [46,51–54] by means of which they provide numerous health benefits including chemoprevention [55], neuroprotection [56], alleviation of obesity and insulin resistance [57]. Therefore, the GLs and their hydrolysis products that also demonstrated strong concentration-dependent luciferase activity in the present study, might be responsible for the observed induction of EpRE mediated gene expression by *M*. *subcordata* methanol extracts and may contribute, at least partly, to justify the various traditional medicinal claims on this plant.

Stachydrine and trigonelline were among compounds identified in *M*. *subcordata*, of which stachydrine was most abundant (**Table 1**). The identification of stachydrine in *M*. *subcordata* supports an earlier study which reported tetramethylammonium, prolinebetaine ethyl ester, stachydrine, 3-hydroxystachydrine and/or 4-hydroxystachydrine (betonicine), 3-hydroxy-1,1-dimethylpyrrolidinium, and glycine betaine in *M*. *subcordata* (*Syn*: *Courbonia subcordata*) [58]. Also, it was shown that stachydrine and 3-hydroxystachydrine along with GLs characterize the family Capparidaceae [58–60]. Trigonelline is a known inhibitor of Nrf2 mediated gene expression [61,62] and results (**Fig 4**) of the present study support these reports. Stachydrine also showed a similar trend and hence both compounds were regarded as inhibitors of EpRE mediated gene expression, although at relatively high concentrations (100 μM). This implies the co-existence of constituents in *M*. *subcordata* methanol extracts with potential competing effects on EpRE induction. i.e. GLs and their hydrolysis products were shown to be inducers while stachydrine and trigonelline, acted as inhibitors.

In line with previous studies that GLs and ITCs are potent activators of Nrf2 [63], EpRE induction by GLs and ITCs in the present study was, at least, ten times more potent than the inhibition by biogenic amines which may imply that in the presence of combined potent inducers and weak inhibitors, the effect of potent inducers may predominate. This could be one possible reason for the fruit, leaf, and seed extracts of *M*. *subcordata* which showed a higher net EpRE induction despite the presence of substantial amount of less potent inhibitors such as stachydrine. In addition, since aromatic ITCs are generally less volatile than aliphatic ITCs [64] relatively stable induction may be expected from the former. Thus, induction of EpRE mediated gene expression was evident by the fruit, leaf and seed extracts containing apparently substantial amounts of glucobrassicin (aromatic glucosinolate) despite the presence of different aliphatic glucosinolates in all extracts including root extract which showed some induction that tend to decline with increasing concentration. The unpredictable kinetics of glucosinolates hydrolysis and variation in volatility of ITCs may also contribute to the fluctuation of EpRE induction with increasing concentration of extracts (**Fig 2B&2C**). Yet, the multicomponent and complex nature of the extracts is a great challenge to make sufficient justification because the complexity of plant extracts may be very problematic in maintaining assay integrity. Cell based assays with plant extracts often fail to fulfil assay requirements like high reproducibility, accuracy, and robustness as plant extracts may contain interfering compounds that co-exist with bioactive compounds [65]. Such possible interference was highly reflected by the leaf extract which showed high variability in cytotoxicity and induction of EpRE-mediated gene expression even at the same concentration assayed in different times. Despite these challenges the present study revealed the intrinsic potential of *M*. *subcordata* extracts to exert potential beneficial effects *via* induction of EpRE-mediated gene expression.

To sum up, the present study demonstrated that *M*. *subcordata* contains bioactive metabolites typically glucosinolates and biogenic amines which are considered important nutraceutical agents and hence the plant may be a good sources of a noble food product. The glucosinolartes are known inducers of antioxidant and detoxifying enzymes while some of the biogenic amines demonstrated inhibitory effect which may imply that process manipulation of the plant is required to get enriched inducer or inhibitor product. Under assay conditions of possible additive and/or antagonistic effects and complex kinetic interactions among multicomponent extracts, the fruit, leaf, and seed extracts revealed a net strong induction of EpRE mediated gene expression while the root extract showed a net absence or weak induction.

## Conclusion

This study is the first to demonstrate the EpRE mediated gene expression induction capacity of *M*. *subcordata*. Especially, the fruit, leaf, and seed extracts exhibited substantial induction while the induction by the root extract was less than twofold at 30 gDW/L. LC-MS^n^ metabolic profiling of the plant revealed the presence of glucosinolates, which are known inducers, and some biogenic amines such as stachydrine and trigonelline that demonstrated as inhibitors. EpRE inducers are cytoprotective and potential chemopreventive agents while inhibitors are suggested adjuvants of chemotherapy implying that processing manipulation of this plant may result in herbal preparations or functional food products that may be used as chemopreventive agents or adjuvants of chemotherapies.

## Supporting information

S1 Fig Map of the plant collection area. (A) location of Tigray region (shaded green), in Ethiopia (B) location of the district (shaded blue) in Tigray, and (C) the localities of plant collection.
(TIF)

S2 Fig Glucosinlates tentatively identified in *M. subcordata* methanol extracts as taken from the display on MAGMa interface. (TIFF)

S3 Fig Some biogenic amines tentatively identified in *M. subcordata* methanol extracts as taken from the display on MAGMa interface. (TIFF)

## Abbreviations

AREs: antioxidant response elements
DMEM/F12: Minimum Essential Medium alpha 1:1 mixture of Dulbecco’s modified Eagle’s medium and Ham’s F12 medium
DMSO: dimethyl sulfoxide
EpRE: electrophile-response element
FCS: foetal calf serum
gDW/L: gram dry weight per litre
GLs: Glucosinolates
LC−MS^n^: Liquid chromatography (LC) coupled with multistage mass spectroscopy (MS^n^)
MAGMa: 'Ms Annotation based on in silico Generated Metabolites'
NEAA: nonessential amino acids
Nrf2: nuclear factor erythroid 2-related factor 2
PBS: Phosphate Buffer Saline
tBHQ: tert-butylhydroquinone
α-MEM: Minimum Essential Medium alpha
ISO: International Organization for Standardization
