# Supplementary figures and images for: Effects of Maerua subcordata (Gilg) DeWolf on electrophile-responsive element (EpRE)-mediated gene expression in vitro
Source: PLoS One. 2019 Apr 15;14(4):e0215155. doi: 10.1371/journal.pone.0215155 (PMC6464171; doi:10.1371/journal.pone.0215155)

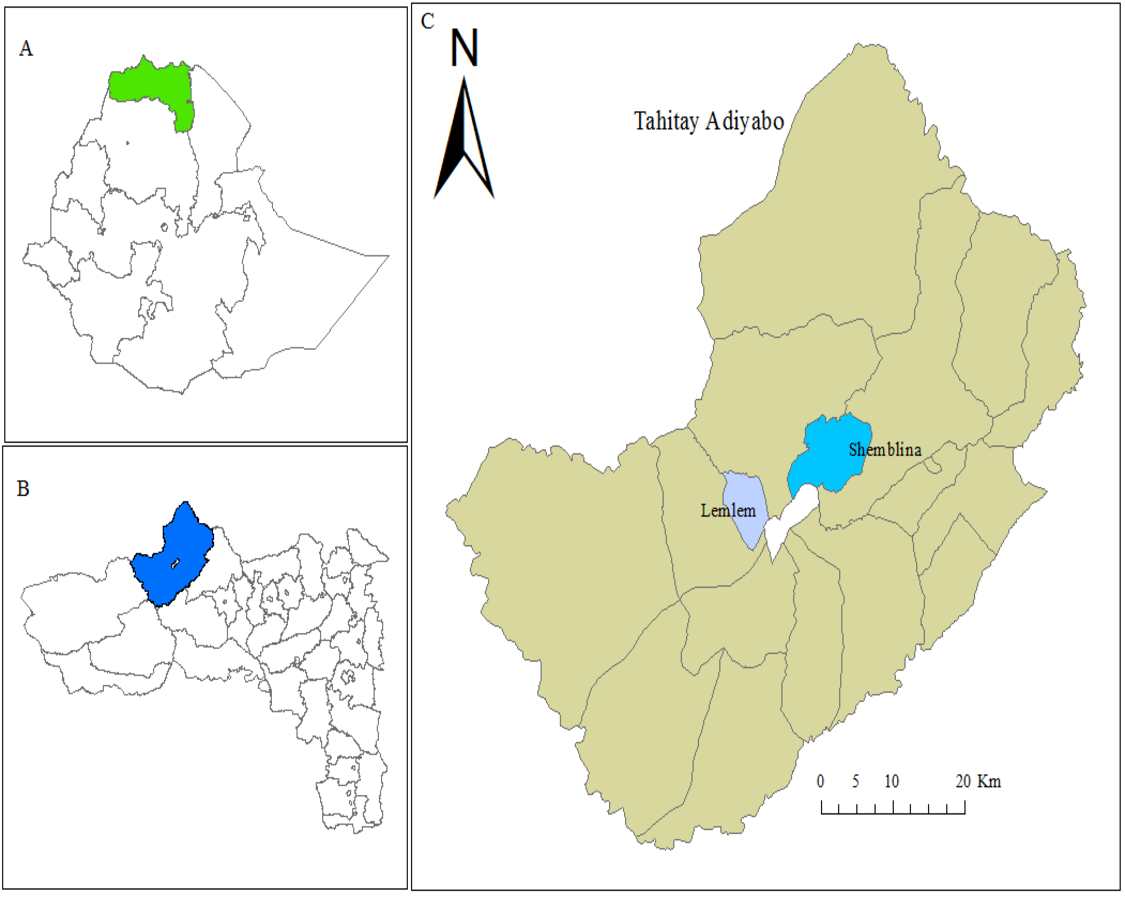

Supplement: S1 Fig — (A) location of Tigray region (shaded green), in Ethiopia (B) location of the district (shaded blue) in Tigray, and (C) the localities of plant collection. (TIF) [file pone.0215155.s001.tif]

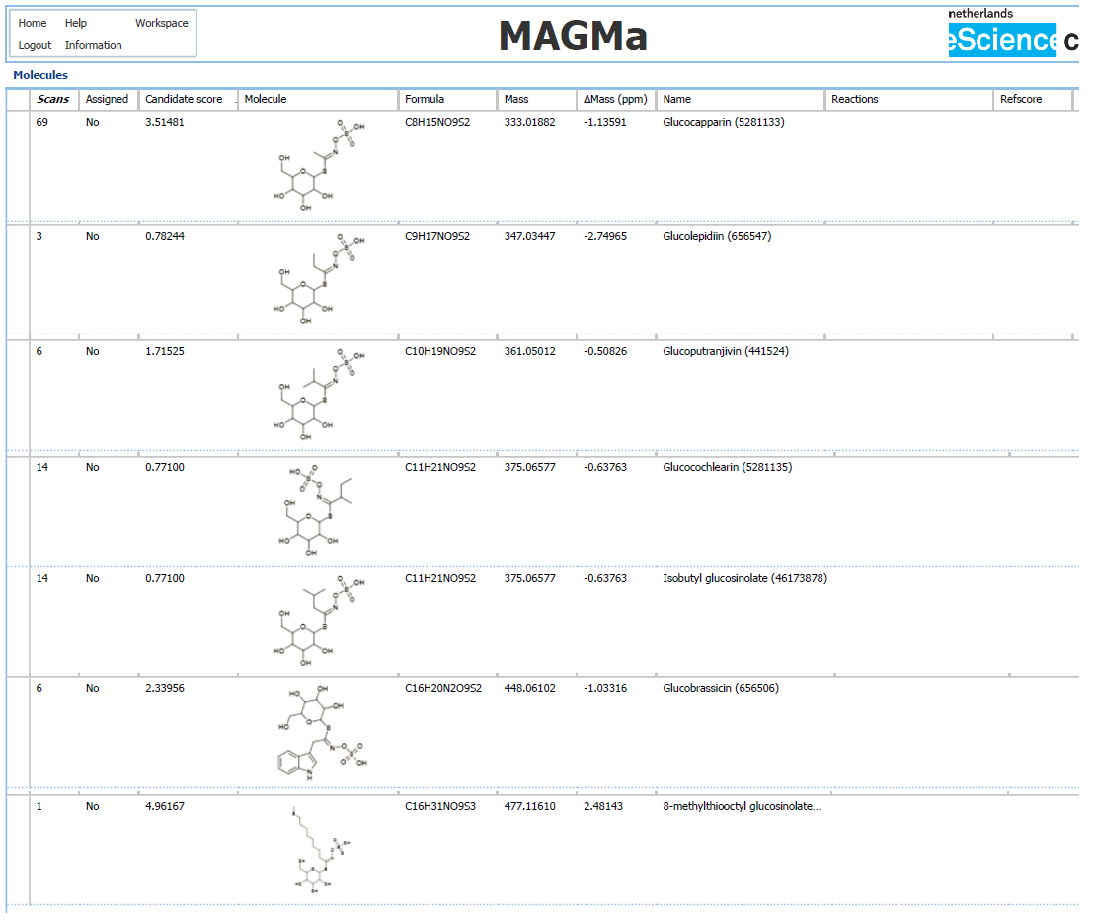

Supplement: S2 Fig — (TIFF) [file pone.0215155.s002.tiff]

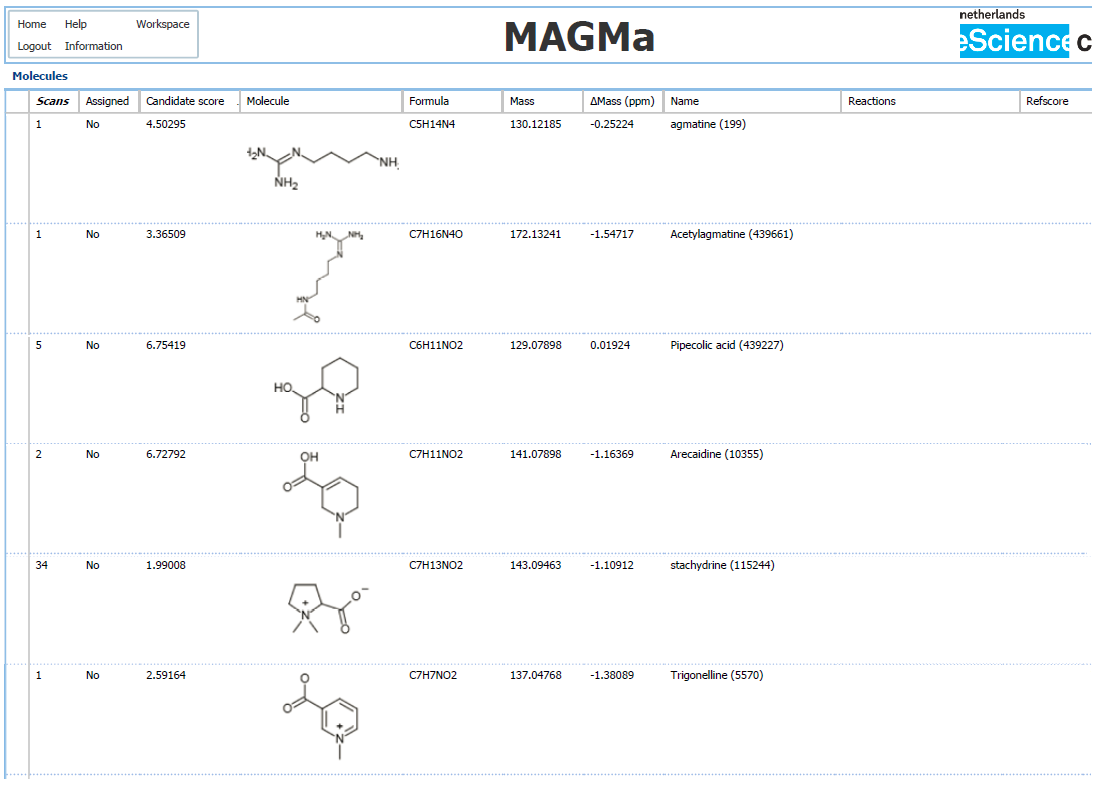

Supplement: S3 Fig — (TIFF) [file pone.0215155.s003.tiff]
